# A high throughput zebrafish chemical screen reveals ALK5 and non-canonical androgen signalling as modulators of the *pkd2*^−/−^ phenotype

**DOI:** 10.1038/s41598-019-56995-7

**Published:** 2020-01-09

**Authors:** A. Metzner, J. D. Griffiths, A. J. Streets, E. Markham, T. Philippou, F. J. M. Van Eeden, A. C. M. Ong

**Affiliations:** 1 Kidney Genetics Group, Academic Unit of Nephrology, Department of Infection, Immunity and Cardiovascular Disease, University of Sheffield, Sheffield, UK; 2 The Bateson Centre, University of Sheffield, Sheffield, UK; 3 Department of Biomedical Science, University of Sheffield, Sheffield, UK

**Keywords:** Disease model, Polycystic kidney disease

## Abstract

Autosomal dominant polycystic kidney disease (ADPKD) is the most common monogenic cause of end-stage renal failure in humans and results from germline mutations in *PKD1* or *PKD2*. Despite the recent approval of tolvaptan, safer and more effective alternative drugs are clearly needed to slow disease progression. As a first step in drug discovery, we conducted an unbiased chemical screen on zebrafish *pkd2* mutant embryos using two publicly available compound libraries (Spectrum, PKIS) totalling 2,367 compounds to identify novel treatments for ADPKD. Using dorsal tail curvature as the assay readout, three major chemical classes (steroids, coumarins, flavonoids) were identified from the Spectrum library as the most promising candidates to be tested on human *PKD1* cystic cells. Amongst these were an androgen, 5α−androstane 3,17-dione, detected as the strongest enhancer of the *pkd2* phenotype but whose effect was found to be independent of the canonical androgen receptor pathway. From the PKIS library, we identified several ALK5 kinase inhibitors as strong suppressors of the *pkd2* tail phenotype and *in vitro* cyst expansion. In summary, our results identify ALK5 and non-canonical androgen receptors as potential therapeutic targets for further evaluation in drug development for ADPKD.

## Introduction

Autosomal Dominant Polycystic Kidney Disease (ADPKD) is one of the most common monogenic diseases with an estimated point prevalence of 1 in 2500 and a genetic prevalence of 1 in 1000^1,2^. It is the fourth most common cause of end-stage renal disease (ESRD) affecting 10–12 million people world-wide. In ADPKD, the diseased kidneys contain numerous fluid-filled cysts which grow to destroy normal kidney tissue. The major risk factors for worse kidney outcome include genotype, age, gender and total kidney volume (reviewed in^1^). The vast majority of patients with ADPKD carry germline mutations in *PKD1* or *PKD2*. Regarding ESRD, a clear genotype-phenotype correlation has been demonstrated, patients with *PKD1* truncating mutations reaching ESRD earlier than those with *PKD2* mutations and those with *PKD1* non-truncating mutations having an intermediate phenotype^3,4^. Total kidney volume (TKV) adjusted for age and height is also a strong predictor of renal prognosis^5^. Finally, it has been observed that male patients typically reach ESRD earlier than females and have larger kidneys^3,6^.

To date, only one drug (tolvaptan) has been approved to treat ADPKD patients with a high risk of disease progression^7^. However, it is only moderately effective and is associated with a high incidence of poorly-tolerated side effects mainly due to increased aquaresis^8^. Monthly monitoring of liver function is also mandated in all countries where tolvaptan has been licenced due to idiosyncratic liver toxicity in the pivotal trials.

The ADPKD proteins PC1 (polycystin-1) and PC2 (polycystin-2) are thought to form a plasma membrane receptor-ion channel complex^9,10^. The exact functions of the PC1-PC2 complex remain unclear, although mutations in either lead to altered Ca^2+^ and cAMP signalling^11^. Nonetheless, changes in a range of other signalling pathways and cellular functions have been reported^12^. PC2 has been localised in multiple cellular compartments including primary cilia, apical and basolateral membranes, endosomes, mitochondria and the endoplasmic reticulum where it could mediate PC1-independent functions in intracellular Ca^2+^ regulation^10,13–15^.

The high degree of sequence conservation between zebrafish *pkd2* and human *PKD2* (67% identity) has stimulated several groups to use zebrafish *pkd2* morphants as a model to study ADPKD^16–18^. A striking characteristic feature of all reported *pkd2* morphants and mutants is a profound upward tail curvature at 40 hpf (hence the further denomination of the *pkd2* mutant as *curly up* (*cup)* from when it was first isolated from a phenotype-based screen^19^). This contrasts with zebrafish ‘cilia’ mutants where conversely there is downward tail curvature^19–21^. Unlike *pkd2* mutants, *pkd2* morphants develop cystic kidneys, although this appears to be restricted to the glomerulus and proximal tubules rather than the entire pronephros like in other cystic cilia mutants^16,17,22^. Obara *et al*. suggested that a partial cloacal occlusion in *pkd2* could lead to glomerular dilatation secondary to a build-up of fluid^16^.

The utility and advantages of using zebrafish embryos as a model for *in vivo* chemical library screening has been exploited by many groups for non-renal diseases^23,24^ although a large unbiased chemical screen for ADPKD has not been previously reported. The potential for such an approach however has been shown by a previous study using a small focussed library in *pkd2* mutants^25^. Here, an inhibitor of histone deacetylase (HDAC) identified from their screen was subsequently also tested in several *Pkd1* mouse models and found to inhibit disease progression^26,27^. In this paper, we report the results of a high throughput chemical library screen in *pkd2* mutant zebrafish using tail curvature as a phenotypic readout. Combined with validation assays in mammalian cyst assays, this approach allowed us to identify two new pathways of potential relevance for future drug development i.e. ALK5 (Transforming growth factor beta receptor I) and non-canonical androgen signalling.

## Results

### Development of a strategy for chemical library screening in zebrafish embryos

**Figure 1:**
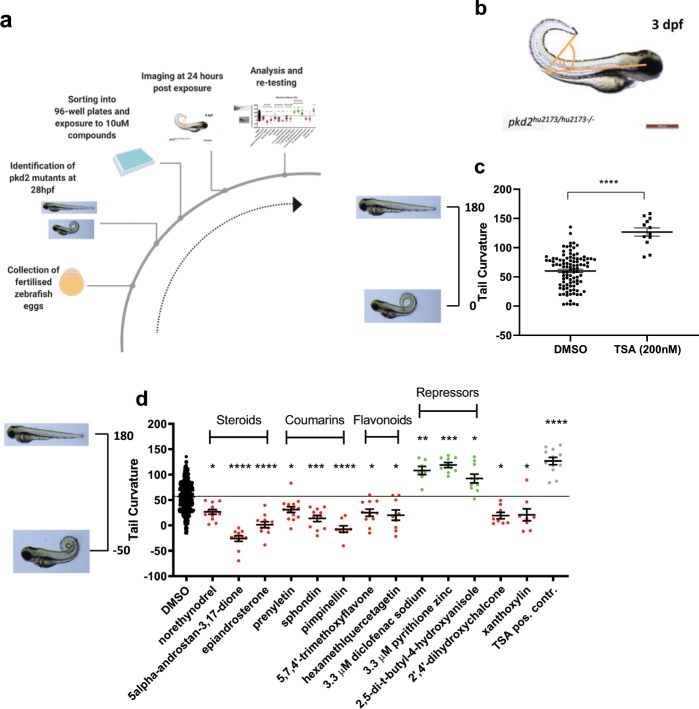
A high through-put zebrafish screen of *pkd2*^−/−^ phenotype modulators. (**a**) Workflow of compound screen on *pkd2* tail curvature phenotype. (**b**) *pkd2*^−/−^ embryo at 3 dpf with schematic indication of curvature measurement. 180° - straight tail, 0° - tail crosses body axis. (**c**) Curvature analysis of *pkd2* mutants exposed from 27 hpf to DMSO or 200 nM TSA. Example images of curvature next to y-axis. Significance via Mann-Whitney test; ****p ≤ 0.0001. (**d**) Combined data on hit compounds of the Spectrum library after initial compound screen, validation and cherry-picked compound exposures. Enhancers of *pkd2* curvature in red, repressors in green. Representative tail curvature images on the left of results. Chemical classes as indicated. Mean of DMSO baseline indicated with black line. Significances via Kruskal-Wallis test; ****p ≤ 0.0001, ***p ≤ 0.001, **p ≤ 0.01, *p ≤ 0.05 and non-significant (ns): p > 0.05. (**a**) created using BioRender.com.

The strategy we adopted is shown schematically in Fig. 1. We initially explored the possibility of using morpholino-induced *pkd2* knockdown, to utilise both glomerular dilation and tail curvature as independent readouts for a chemical library screen in zebrafish embryos, as has been previously reported^25^. However, in our hands, morpholino injections were associated with low and variable penetrant cyst formation as well as highly variable tail curvature. Successful high-throughput screens require robust and fully penetrant effects, especially where the aim is to identify phenotype suppressors. We therefore decided to use a TILLING ENU zebrafish mutant generated previously i.e. *pkd2*^*hu2173/hu2173*^ (henceforth referred to as *pkd2*^−/−^) using tail curvature (curly-up) as a quantitative readout (Fig. 1b), as previously reported in a small-scale screen^25^. Dechorionated mutant embryos were sorted at 29 hpf, based on a slight curvature of the tail prior to full maturation of the phenotype by 48 hpf. As proof-of-principle, trichostatin A (TSA) was used as a positive control^25^ to demonstrate that it was possible to sort the mutants just prior to chemical exposure and still successfully suppress the tail curvature phenotype (Fig. 1c). This allowed us to expose recessive *pkd2* mutants to individual compounds rather than using larger groups of embryos with a reduced fraction (25%) of mutant embryos.

Taking this approach, we conducted a chemical screen first using the Spectrum library which contains a diverse set of 2000 bioactive compounds including FDA-approved drugs^28^. After initial testing at a concentration of 10 µM, 200 compounds of interest were identified (see Materials and Methods) and after 2 further retests, 13 compounds remained that significantly and reproducibly altered the curvature phenotype: 10 compounds enhanced and 3 repressed the curvature (Fig. 1d). Of interest, 8 of the enhancers clustered into 3 chemical classes: steroids, coumarins, and flavonoids. Further details of all the hit compounds identified can be found in Supplementary Information (Table S1).

### Identification of non-canonical androgen signalling as a modulator of the *pkd*2 phenotype

**Figure 2:**
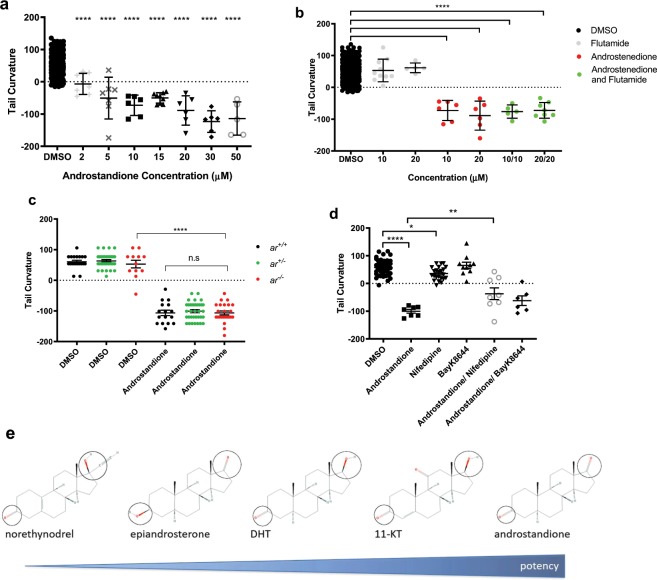
Androgens modulate tail curvature in *pkd2*^−/−^ embryos independently of AR. (**a**) Dose-response effects of androstandione on tail curvature in *pkd2*^−/−^ zebrafish embryos. (**b**) Effects on tail curvature in *pkd2*^−/−^ zebrafish embryos with co-exposure of androstandione and the anti-androgen flutamide. (**c**) The effects of androstandione on tail curvature in *pkd2*^−/−^;*ar*^−/−^ embryos. (**d**) Effects of co-exposure of androstandione (10 µM) and the LTCC inhibitor, nifedipine (10 µM) or agonist, BayK8644 (30 µM), on tail curvature of *pkd2*^−/−^ zebrafish embryos. All results represented by mean +/− SEM. Significance via one-way ANOVA with Dunnett’s multiple comparisons. P values represented by * (****p ≤ 0.0001, ***p ≤ 0.001, **p ≤ 0.01, *p ≤ 0.05 and non-significant (ns): p > 0.05.). (**e**) Chemical structures of androgens and their potency on *pkd2*^−/−^ tail curvature with androstandione being the most potent and norethynodrel the least potent. Circles are positioned at C3 and C17, with the complexity of side groups at these locations correlating with potency of the compound.

The most potent tail curvature enhancer identified was 5α-Androstan-3,17-dione (CAS No. 846-46-8, henceforth referred to as androstandione). Given that male ADPKD patients develop larger kidneys and develop ESRD earlier than female patients^3,29,30^, we decided to study this compound further and investigate the role of androgens more extensively. Surprisingly, androstandione proved to be more potent in this assay than other androgens including dihydrotestosterone (DHT), the most potent human androgen^31^, and 11-ketotestosterone (11-KT), the most potent fish androgen^32^. It enhanced dorsal tail curvature from 2 µM (lowest concentration tested) to 30 µM, where all treated embryos had tails that curled well beyond the body axis (Fig. 2a). In comparison, DHT and 11-KT only significantly altered tail curvature at 50 µM and 30 µM respectively (Fig. S1). Norethynodrel, a synthetic progesterone, was also identified from the screen (Fig. 1d). It is rapidly metabolised into α- and 3β-OH-NOR^33^, neither of which are well characterised. However, oral administration of norethynodrel has been associated with progesterone receptor activation as well as weak androgenic activity^34^. Differences in the chemical structure and potency of the different androgens tested in this assay are shown in Fig. 2e.

We next examined the ability of the androgen receptor (AR) antagonist flutamide to inhibit the effect of androstandione. As shown, flutamide did not inhibit the tail curvature induced by androstandione nor did it have an independent effect (Fig. 2b). These results raised the possibility that androstandione could be acting via an AR-independent pathway.

To prove that this was the case, we took a genetic approach by generating *pkd2* mutant fish lacking the *ar* gene by CRISPR/Cas mutagenesis. A CRISPR sequence designed to mutate the *ar* gene was injected into *pkd2/*+embryos, G_0_ carriers were raised and the *sh516* allele was isolated. This allele contains a 2 bp deletion (GC) at codon 453 of the zebrafish *ar* gene, leading to a frameshift and a premature stop after the addition of 15 novel aa in the AR protein. We confirmed that this mutation resulted in loss of secondary sex characteristics (such as anal fin colour) in males but preservation of the withdrawn external genital papilla (Fig. S2a). Histological sections demonstrated significantly distorted and disorganised testicular structures which lacked large spermatozoa pools in *ar* mutants (Fig. S2b). These features are consistent with previously reported *ar* knockout zebrafish mutants^35–37^. *pkd2/* + ;*ar/* + fish were then bred and the embryos treated with androstandione or vehicle (DMSO) to assess the effect on tail curvature. Loss of *ar* did not alter curvature enhancement by androstandione nor had an independent effect on the curvature phenotype (Fig. 2c).

A number of rapid, plasma membrane located androgen signalling pathways independent of the AR have been identified over the last decade^38^. One such pathway is the direct inhibitory action of androgens on L-type calcium channels (LTCC)^39–43^. Of interest, LTCC subunits (Ca_v_1.2) are reported to be expressed in primary cilia, zebrafish *ca*_*v*_*1.2* morphants exhibit “cystic kidneys” and lentiviral *Ca*_*v*_*1.2* knockdown in *Pkd1*^+/−^ mice resulted in severe PKD^44^. To further investigate this possibility, we utilized a potent LTCC antagonist (Nifedipine) and agonist (BayK8644) to assess their ability to modify tail curvature in *pkd2* embryos. As shown (Fig. 2d), BayK8644 had no effect on tail curvature while nifedipine only slightly increased tail curvature. However, nifedipine had a significant effect in counteracting the effect of androstandione although BayK864 was neutral in the same assay (Fig. 2d). Therefore, although it is possible that androstandione could be acting as an inhibitor of LTCC, this is likely to be a minor pathway.

To investigate the role of other non-canonical androgen signaling pathways, we interrogated published microarray data of differential gene expression in cystic and non-cystic cell lines^45^. There was no difference in expression in any of the four LTCC subunits or other potential non-canonical androgen receptors (Fig. S3). Further analysis was performed in microarray data derived from normal (non-ADPKD) and ADPKD human kidneys, the latter discriminating between minimally cystic tissue (MCT), small, medium and large cysts^46^ (see Materials and Methods). There was significantly reduced expression of ZIP9 and the LCC subunit CaV1.2 in all cysts from human ADPKD kidneys (Fig. S4).

### A second *pkd*2 zebrafish screen using the published kinase inhibitor set (PKIS) library

**Figure 3:**
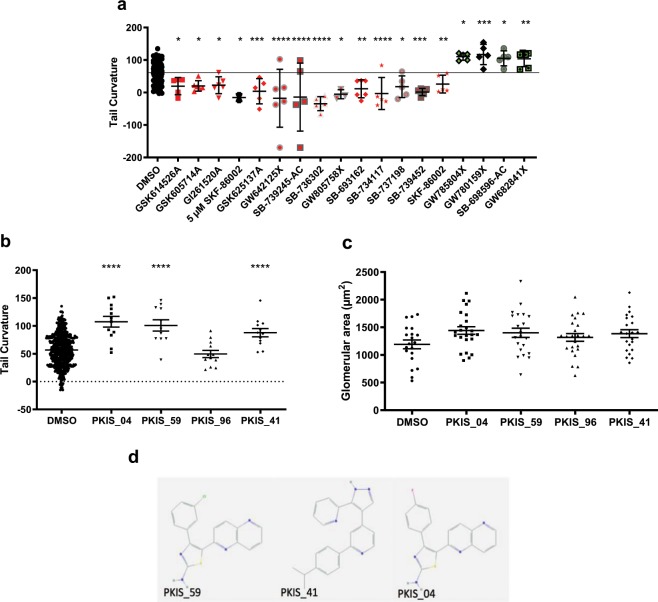
PKIS zebrafish screen revealed four PKIS repressors of the *pkd2*^−/−^ phenotype. (**a**) Hit compounds identified from the PKIS library after initial compound screening and validation. Enhancers of *pkd2* curvature in red, repressors in green. Mean of DMSO baseline indicated with black line. Effect of re-ordered PKIS repressor compounds on *pkd2* curvature (**b**) and glomerular area (**c**). Statistical significances by one-way ANOVA with Dunnett’s multiple comparison, results represented by mean +/− SEM. P values represented by * (****p ≤ 0.0001, ***p ≤ 0.001, **p ≤ 0.01, *p ≤ 0.05 and non-significant (ns): p > 0.05.). (**d**) Chemical structure of the three validated PKIS tail curvature repressor compounds.

Using the same strategy, we conducted a second chemical library screen using the Published Kinase Inhibitor Set (PKIS) set of 367 kinase inhibitors to identify additional repressor compounds^47^. 18 consistently active compounds were identified with the majority enhancing tail curvature as in the Spectrum compound screen (Fig. 3a). Because of their potential applicability for drug development, we decided to focus on the four identified repressor compounds and their respective kinases: GW785804X (PKIS_04), GW780159X (PKIS_59), SB-698596-AC (PKIS_96) and GW682841X (PKIS_41).

Confirmatory testing with new batches of chemicals replicated these findings except for PKIS_96 which we therefore discarded (Fig. 3b). Glomerular diameter was also assessed using *pkd2*^*hu2173/hu2173*−/−^*;wt1b:GFP* embryos since GFP expression driven by the Wilms tumour 1b (Wt1b) promoter allows visualisation of the pronephros: glomerular diameter was unaffected by any of the four PKIS repressor compounds (Fig. 3c).

Based on published information, PKIS_04 and PKIS_59 are known to inhibit KDR (kinase insert domain receptor)and PKIS_41 inhibits MAP4K4 (mitogen-activated protein kinase kinase kinase kinase 4)^48^. As shown, PKIS_04 and PKIS_59 have a very similar chemical structure (Fig. 3d). In addition, we noted that three PKIS repressor compounds were initially designed to inhibit ALK5 (Table S2).

### Validation of spectrum and PKIS compounds in mammalian cyst assays

Next, we tested the ability of our chemical hits to modify cyst formation in 3D assays. Two cell lines were used: MDCKII cells^49,50^ and a human-derived *PKD1* cystic cell line (OX161c1)^45,51^. Initial pilot experiments were conducted over a range of concentrations to exclude cell toxicity at the doses tested.

**Figure 4:**
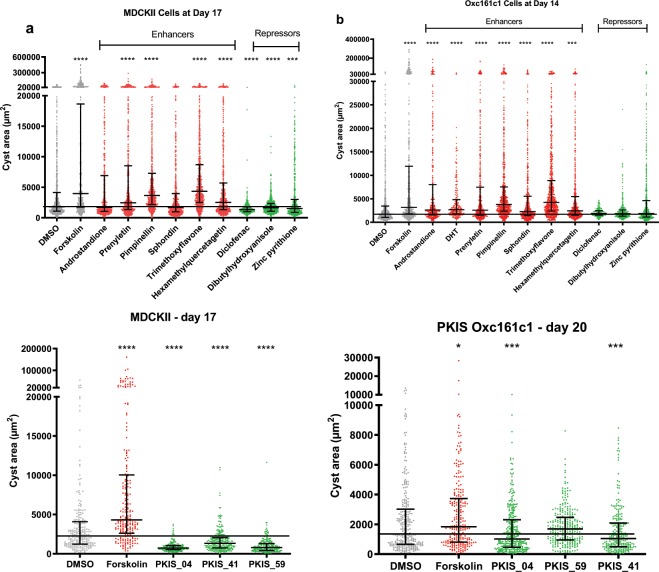
Chemical compounds and PKIS inhibitors identified from zebrafish screen in two cystic cell culture models, MDCKII and Ox161c1. Cyst area of MDCKII (**a**) and Ox161c1 (**b**) cells after 17 and 14 days of compound exposure respectively to compounds identified in the zebrafish screen. Chemical classes as indicated, concentrations determined via prior dose response assays to exclude toxicity. Cyst area of MDCKII (**c**) and Ox161c1 (**d**) cells after 17 and 20 days of compound exposure respectively to compounds identified in the zebrafish screen compared to DMSO control exposed cells. PKIS inhibitors as indicated at 10 *µ*M concentrations and 5 µM forskolin. Results represented by median +/− IQR. P values represented by * (****p ≤ 0.0001, ***p ≤ 0.001, **p ≤ 0.01, *p ≤ 0.05 and non-significant (ns): p > 0.05.). Median of DMSO baseline indicated with black line and significances via Kruskal-Wallis test with Dunn’s multiple comparison.

Overall, the results in both cell assays for the Spectrum library compounds showed similar results to those predicted from the zebrafish screens i.e. enhancers increased cyst area and repressors reduced cyst area, although statistical significance was not reached for all compounds from two independent replicate experiments due to variability (Fig. 4a,b). There were, however, several differences between the lines: androstandione had a more potent effect in OX161c1 than MDCKII but the converse was true for the three repressor compounds: the three repressor compounds from the PKIS library had a more pronounced in the MDCKII cells at the concentrations tested (Fig. 4c,d). These could reflect cell type or species differences.

To further assess the efficacy of our identified repressor compounds, we tested their effects in the presence of Forskolin. Forskolin is a known agonist of adenylate cyclase and has potent cystogenic effects. The results were consistent between the two cell lines with diclofenac the most potent repressor and zinc pyrithione, the least (Fig. S5).

### Refining the target kinase to ALK5

**Figure 5:**
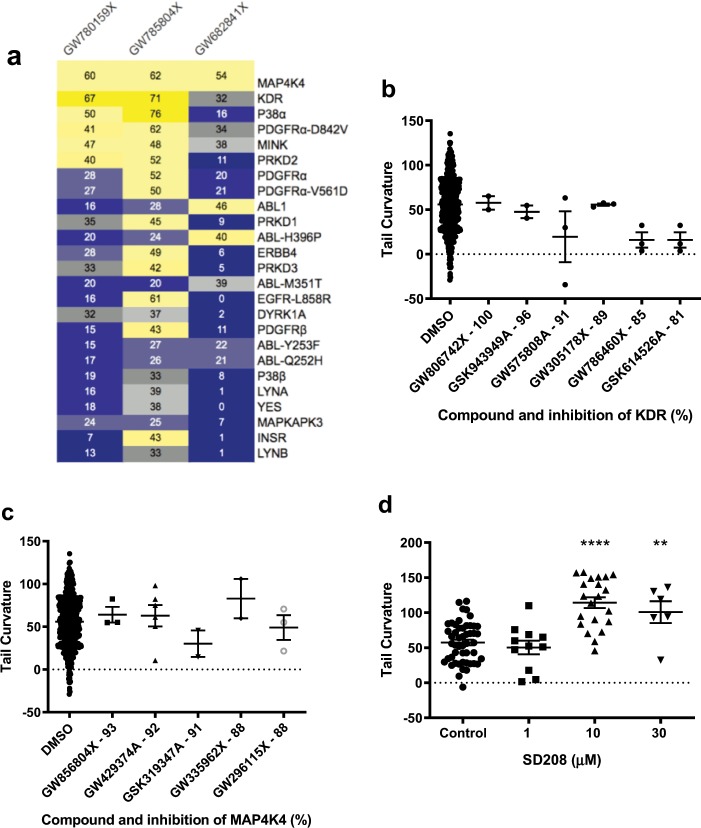
Identification of ALK5 as a *pkd2*^−/−^ phenotype modulator in zebrafish. (**a**) Heat maps of known kinases inhibited by PKIS repressor compounds with the top 25 inhibited kinase targets in decreasing order. Numbers represent percentage of inhibition; dark yellow ≥65%, yellow ≥40%, light grey 35–40%, dark grey 30–35%, grey/blue 20–30%, light blue 15–20% and dark blue >15%. (**b**) Effects of potent KDR and (**c**) MAP4K4 inhibitors on *pkd2*^−/−^ zebrafish embryo tail curvature. All compounds tested at 10 µM. Results represented by mean ± SEM. No significance via one-way ANOVA and Dunnett’s multiple comparison. (**d**) Effects of different concentrations of the ALK5 inhibitor SD208 on *pkd2*^−/−^ curvature. Results represented by mean ± SEM. Significance via one-way ANOVA and Dunnett’s multiple comparison, p values represented by * (****p ≤ 0.0001, ***p ≤ 0.001, **p ≤ 0.01, *p ≤ 0.05 and non-significant (ns): p > 0.05).

As shown on the heat map (Fig. 5a) of potential kinases targeted by PKIS_04, 41 and 59, we identified Kinase insert domain receptor (KDR) as the most relevant target for PKIS_04 and _59 whilst MAP4K4 was the most prominent kinase when all three repressors were combined. However, other potent inhibitors of KDR (Fig. 5b) and MAP4K4 (Fig. 5c) present in the library on tail curvature had no effect on tail curvature, suggesting that neither kinase was likely to be relevant in this assay.

We noted that PKIS_04 and PKIS_59 were initially designed alongside PKIS_41 to inhibit ALK5 (also known as TGFßR1 - transforming growth factor, beta receptor I) (Table S2). Although ALK5 was not amongst the original kinases tested^48^ in the initial characterisation of the PKIS compound collection, ALK5 inhibiting properties for these compounds have been reported^52,53^. To confirm that ALK5 was indeed the relevant kinase targeted, we tested the effect of a structurally-unrelated ALK5 inhibitor (SD208) with higher specificity^54^. As shown, SD208 had a profound effect in repressing tail curvature in *pkd2* embryos at two concentrations (10 and 30 μM) (Fig. 5d).

**Figure 6:**
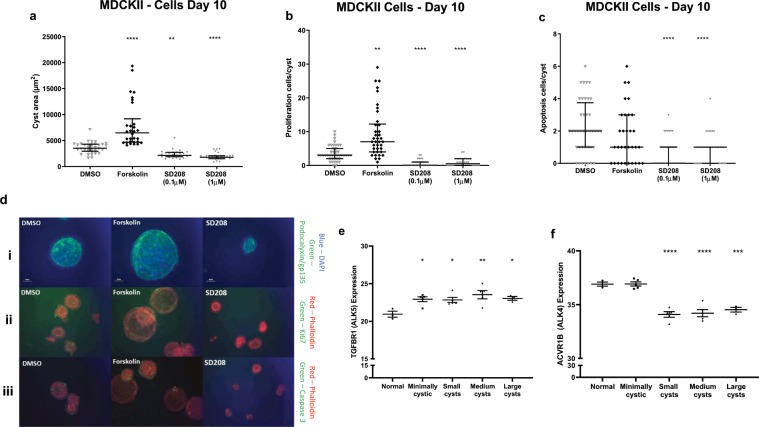
ALK5 as a modulator of cystic growth in cell culture and expression of ALK4/5 in human ADPKD kidneys. Effects of SD208 (1 and 0.1 µM) after 10 days exposure on cyst area (**a**), proliferation (**b**) and apoptosis (**c**) in MDCKII cells compared to DMSO and forskolin controls. Results represented by median +/− IQR. Significance via Kruskal-Wallis test with Dunn’s multiple comparison, p values represented by * (****p ≤ 0.0001, ***p ≤ 0.001, **p ≤ 0.01, *p ≤ 0.05 and non-significant (ns): p > 0.05.). (di, ii, iii) Representative images of SD208 effects on cyst area, proliferation and apoptosis respectively compared to DMSO or forskolin. Podocalyxin/gp135 was used to define lumen formation with nuclear DAPI marker (di), Ki67 and cleaved caspase 3 antibodies used to measure proliferation and apoptosis respectively with phalloidin apical markers (dii and diii). Microarray expression profile of ALK5 (**e**) and ALK4 (**f**) in human kidney tissues derived from 5 polycystic kidney samples and 3 non-polycystic kidney samples. Polycystic sample tissues are separated into minimally cystic, small cysts (<1 ml), medium cysts (10–20 ml) and large cysts (>50 ml). Results represented by mean +/− SEM. Significance via one-way ANOVA with Dunnett’s multiple comparison, p values represented by * (****p ≤ 0.0001, ***p ≤ 0.001, **p ≤ 0.01, *p ≤ 0.05 and non-significant (ns): p > 0.05).

In MDCK cysts, suppression of cyst growth was observed at even lower concentrations of SD208 (0.1 and 1 μM) (Fig. 6a). The inhibitory effect of this ALK5 inhibitor was associated with a significant suppression of proliferative (Ki67 positive, Fig. 6b) and apoptotic (cleaved caspase-3 positive, Fig. 6c) cell number without an obvious effect on lumen formation (podocalyxyn or gp135, Fig. 6d). These results lead us to conclude that ALK5 activity has a stimulatory role in cyst growth.

To confirm our findings, we again interrogated a published microarray of differential gene expression in normal (non-ADPKD) and ADPKD human kidneys^46^. ALK5 expression was significantly increased even in MCT and cysts whereas ALK4 expression was unchanged in MCT but significantly decreased in all cysts (Fig. 6d,e). SMAD2 expression was significantly increased in all cysts and SMAD3 expression increased in large cysts (Fig. S6).

## Discussion

In this paper, we report the results of the first unbiased chemical library screen conducted in a zebrafish model of ADPKD using tail curvature of *pkd2*^−/−^ embryos as a phenotypic readout. Positive hits (both enhancers and repressors) were then confirmed in 3D cyst assays using two cell lines, one being a well characterised human *PKD1* patient-derived cystic cell line. Our approach identified a number of potent modulators of cyst growth including two major signalling pathways: androstandione acting via AR-independent signalling and ALK5-mediated signalling.

The use of tail curvature as a phenotypic readout for the screens has been previously reported in a smaller focussed library screen^25^. To our knowledge however, this is the first report of a larger unbiased chemical screen using a *pkd2* zebrafish model. We had initially planned to use glomerular dilatation as the primary readout but were unable to do so due to the lack of this feature in the mutants and its inconsistency in MO treated fish. The absence of pronephric cysts or dilatation in several *pkd2* mutant strains has been reported by other groups without a clear explanation^19,22,55^, although Schottenfeld *et al*. have reported that maternal RNA could explain this observation^22^. To exclude compensatory effects of maternal *pkd2* mRNA in the embryos, we injected low doses of an ATG-MO but did not observe pronephric dilatation (Fig. S7). In comparison, *pkd1* mutant fish have been reported to develop pronephric cysts with full penetrance (100% at 3 dpf for *pkd1a/b* double knockouts) although an earlier paper reported a lower frequency (10–20%) with MO knockdown^56,57^.

Although the ‘curly-up’ tail phenotype of *pkd2* mutants and morphants is consistent and well described, its molecular basis is not well understood. The most likely explanation proposed is an increase in extracellular matrix deposition due to an increase in the production and/or deposition of collagen IIα2^57^. Early changes in ECM deposition have been observed in the ADPKD kidney and are likely to be a component of the multiple and complex mechanisms underlying cystogenesis^58,59^.

Three chemical classes could be defined from the compounds identified from the Spectrum library: steroids, coumarins and flavonoids (Table S1). Of interest, the COX-2 inhibitor, diclofenac, was identified as one of three repressors. Preliminary evidence that PGE2 signalling may play a role in ADPKD has been reported especially in cellular assays^60,61^. We were able to confirm the repressor effect of the HDAC inhibitor, TSA, on tail curvature, as previously reported^25^.

**Figure 7:**
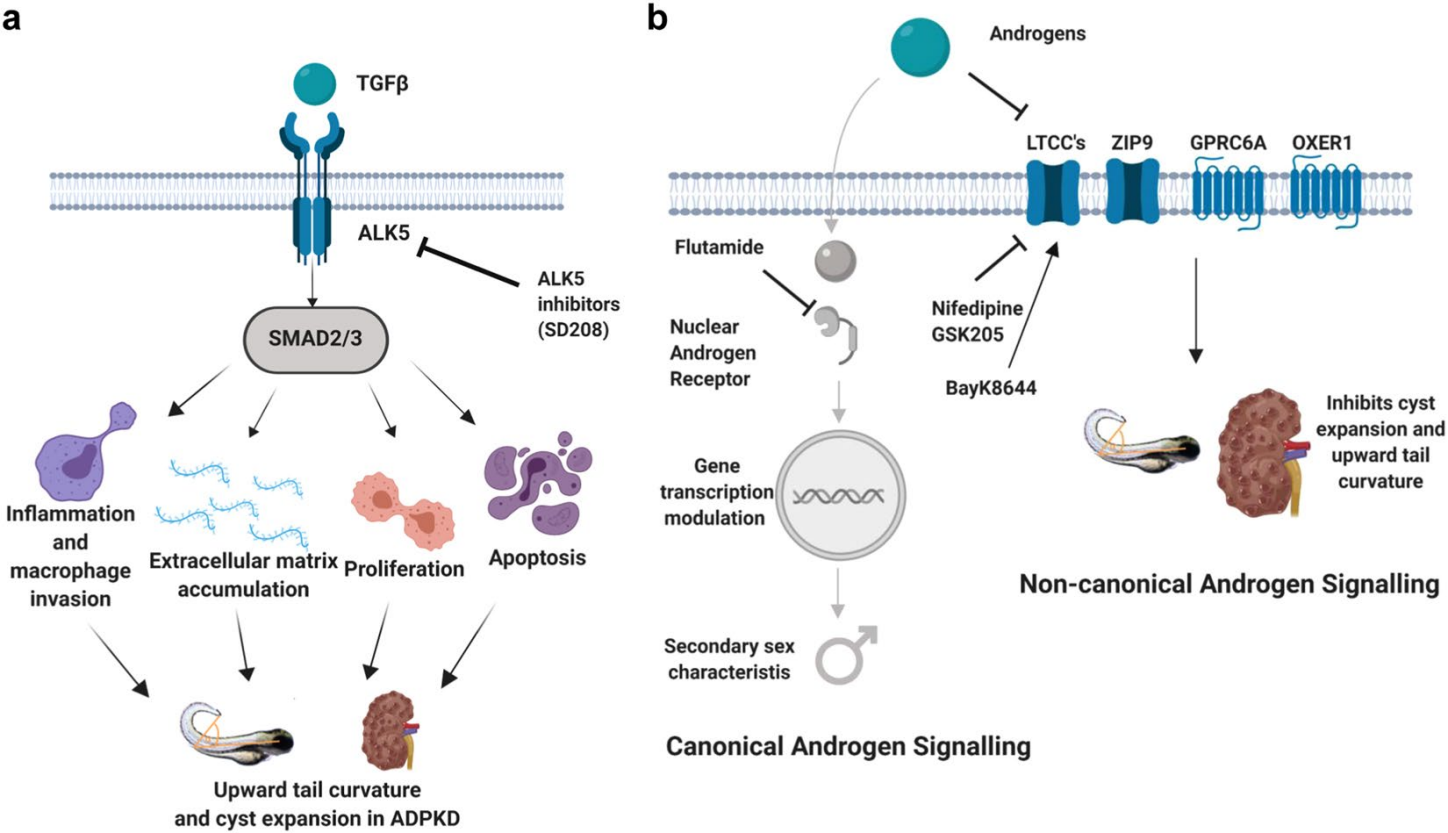
Model for role of ALK5 and non-canonical androgen signaling in ADPKD. (**a**) TGFβ binds to ALK5 (TGFβ-receptor 1) or ALK4 (not shown) at the plasma membrane to activate the transcription factors SMAD2/3. SMAD2/3 in turn activate other TGFβ regulated genes to promote cyst expansion and disease progression by effects on inflammation, extracellular matrix accumulation, proliferation and apoptosis. (**b**) Androgens bind to androgen receptors (AR) in the cytoplasm resulting in translocation of the complex to the nucleus. The AR-androgen complex modulates gene transcription of androgen related genes in the canonical signaling pathway. Androgens can also bind to and inhibit non-genomic receptors such as L-type calcium channels, ZIP9, GPRC6A or OXER1 in a non-canonical, AR-independent pathway. Androgen activation of this non-canonical pathway promotes cyst expansion and upward tail curvature. Figure created with BioRender.com.

Two out of three compounds identified in the steroid group were androgens. The most potent effect of all the enhancers was observed with androstandione and it should be noted that both epiandrosterone and DHT can also be converted to androstandione^62,63^ (Fig. 2e). The much higher potency of androstandione compared to DHT or 11-KT as well as the inability of flutamide to block its effect led us to conclude that it was acting via a non-canonical androgen signalling pathway. Genetic evidence using a zebrafish *ar* mutant confirmed that this was indeed via AR-independent signalling. Potential candidates for this non-canonical pathway include GPRC6A^64^, ZIP9^65,66^, OXER1^67^ and L-type calcium channels (LTCCs)^39–43^. Testosterone has been shown to bind to these receptors, mediating non-genomic activity which is independent of AR (Fig. 7). Of interest, we found a curvature enhancing effect of the specific LCC inhibitor nifedipine^39^ which also partially ameliorated the effects of androstandione, suggestive of LCC competitive antagonism^41^. Although significant, the effects were of a lower magnitude compared to that of androstandione suggesting that other AR-independent pathways are likely to be involved.

The three kinase repressor compounds identified from the PKIS screen recognised ALK5 or TGFß receptor-1 as the common target. We confirmed these findings both positively and negatively by using a more selective and structurally unrelated ALK5 inhibitor (SD208) and testing the effect of inhibitors to the other putative kinases (MAPK4 and KDR) in the tail curvature assay. Importantly, SD208 also inhibited cyst growth in MDCK cells by inhibiting cell proliferation.

There are two branches of the TGFß family: The ALK5 (TGFßR1)/TGFßR2 receptor complex and the ALK4/activin receptor II b complex - both of which ultimately activate SMAD2/3, transcription factors that are the main effectors of the TGFß pathway. It is well known that TGFß signalling mediates a wide range of downstream effects including cell proliferation, differentiation, apoptosis and extracellular matrix deposition (reviewed in^68^), all of which have been implicated in ADPKD disease progression^69^. Both SMAD2 and 3 are upregulated in a variety of mouse models and in human tissues even at early ADPKD disease stages where they are associated with renal fibrosis and epithelial-to-mesenchymal transition (EMT) processes^70,71^. An overview of the proposed ALK5 dependent pathway in *pkd2* mutant zebrafish is represented in Fig. 7. Given that SD208 has not been reported to inhibit ALK4, it is likely that ALK5 rather than ALK4 is the driver behind the reduction of the ADPKD-related phenotype in zebrafish. In this context, activation of SMAD2/3 by fluid shear stress has been demonstrated in immortalised proximal tubular epithelial cells (PTEC) derived from *Pkd1*^*lox,lox*^ mice^72^. This response was ALK5-dependent and significantly increased in *Pkd1*^−/−^ PTEC cells, suggesting a role for exaggerated ALK5-SMAD2/3 signalling in cystogenesis. ALK5 inhibition has been found to reduce fibrosis and extracellular matrix protein deposition *in vitro*^73^. These observations lend support to our findings that ALK5 inhibition can ameliorate the cystic phenotype.

It has been reported that genetic ablation of TGFß-1 in renal epithelial cells of *Pkd1* mutant mice had no effect of cyst development or on SMAD2/3^74^ implicating ALK4 signalling as the primary driver of TGFβ mediated pathology in ADPKD. These differences could relate to species, model or stage of disease. In support of this, our findings from a published human microarray indicate that ALK5 rather than ALK4 expression is increased even in early disease (MCT) with increased SMAD2 expression detectable in small cysts. Of interest, a recent meta-analysis of human and mouse PKD arrays is consistent with our findings^75^.

In conclusion, from a large unbiased chemical library screen in zebrafish *pkd2* embryos, we have identified AR-independent androgen signalling and ALK5 signalling as potential drivers of disease progression in ADPKD. The link with androgens confirms clinical observations of gender-differences in renal prognosis in ADPKD^3,29,30^, gender dimorphism in non-orthologous PKD rodent models^76^ and the protective effects of castration or oestrogens in males^77^. The link with ALK5 and TGFβ signalling confirms a large body of literature showing changes in ECM in early ADPKD. Both pathways will be the subject of future investigation.

## Materials and Methods

### Zebrafish maintenance

Zebrafish were kept under standard conditions (14 h light/10 dark cycle, temperature 26–28 °C^78^). Rearing occurred in E3 medium (5 mM NaCl, 0.17 mM KCl, 0.33 mM MgSO_4_, 0.33 mM CaCl_2_ and methylene blue) and staging according to^79^. To prevent pigmentation, larvae were treated with PTU (phenylthiourea) beginning from 24 hpf (28 °C) when necessary^80^. Zebrafish lines in this project included, amongst others, LWT (London wild type), AB and *pkd2*^*hu2173*^. All procedures adhered to Home Office legislation.

### High throughput *pkd*2 zebrafish chemical screen

A high-throughput chemical screen was conducted by exposing *pkd2*^−/−^ zebrafish to two chemical libraries i.e. the Microsource Discovery’s Spectrum library of 2000 chemicals and the Published Kinase Inhibitor Set of 367 compounds covering 518 known kinases (PKIS, formerly GlaxoSmithKline and subsequently transferred to the University of North Carolina). Zebrafish embryos were exposed in the following manner:

At approximately 24 hpf chorions were removed with pronase (2 mg/ml for 13 minutes), embryos were washed briefly and transferred back to 28 °C. As the curly tail phenotype does not set in at the same stage in all embryos (onset approx. 27–30 hpf), *pkd2* mutants were sorted into a separate dish containing screening medium (E3 medium, 0.75 × PTU and 1% DMSO) as the curvature became apparent. Three embryos were subsequently transferred in 150 µl screening medium to each well of a 96-well plate and 100 µl prepared compound solution (compounds diluted in screening medium, prepared the day prior and kept at –20 °C) were added to a final concentration of 10 µM. Exposed plates were incubated at 28 °C for 24 hpf before imaging each well with the Ash Phenosight system. This is an automated 96-well plate microscope which takes a single brightfield and GFP fluorescent image of each well allowing image acquisition of a whole plate in 10 minutes. Curvature analysis commenced using ImageJ software with methods previously described^57^. DMSO exposed controls were eventually combined as no significant differences between the majority of experimental days were observed (Fig. S8) and a large control group was established.

Initial hits were chosen via a student’s t-test and re-tested in a second round. Additionally, all compounds were retested where more than one embryo had died in the previous exposure round. Compounds were either re-tested at 10 µM (if it was apparent that one decaying embryo had deprived the others of oxygen needed for development) or 0.3 µM if the compound was toxic, after careful assessment of the images. Final hit compounds were determined using one-way ANOVA analysis combining all data collected. To ensure the validity of our hit compounds, individual groups were compared with DMSO control exposed embryos solely from the same batches (Fig. S9).

Subsequently, hit compounds were re-ordered and re-testing commenced using the same conditions as before, also testing a variety of concentrations. Imaging always commenced between 49–52 hpf (curvature is fully developed by 48 hpf and remains stable). Hit compound sources are listed below in Supplementary Table S1.

### Measurement of glomerular and tubular dilation

To visualise the pronephros, *pkd2*^*hu2173/hu2173*−/−^*;wt1b:GFP* embryos and *wt1b:GFP*-positive siblings were anaesthetised and immobilised in methylcellulose. *Wtb1* codes for Wilms tumour protein WT1b which is expressed in all parts of the developing pronephros from 17hpf^81^. Imaging occurred dorsally and glomerular area or tubular diameter was measured using Image J.

### DNA extraction

DNA extraction from single embryos was necessary to determine the *ar* genotype. Single embryos were dechorionated, placed individually in sterile tubes and 50 μl of embryo digestion buffer (10 mM Tris HCl pH 8, 1 mM EDTA, 0.3% Tween20 and 0.3% NP40) was added. The embryos were subsequently heated for 10 min to 98 °C after which 2 μl proteinase K stock solution (25 mg/ml stock) were added. The embryos were then kept at 55 °C for 3 h, which was followed by an inactivation step for 10 min at 98 °C. 2 μl supernatant was subsequently used for PCR reactions. Primers used to genotype *ar* embryos were GACTCTAACGGCCACTACGG, ACGTTAGGGTACGGATGACG. The CRISPR recognition sequence used to generate the *ar* mutant was CCGCACGAGCAGTGGTACCC.

For adult zebrafish, fin clips were used for sequencing. Fin clips were conducted according to Home Office recommendations and clipped materials were transferred directly into 50 μl fresh base solution (1.25 M KOH and 10 mM EDTA in MilliQ H2O) in a 96-well plate. The removed tissue was then incubated for 30 minutes at 95 °C and the plate vortexed for 5 seconds. Subsequently 50 μl neutralisation buffer (2 M Tris HCl in MilliQ H2O) was added to each sample and the plate vortexed again for 10 seconds. Lastly, the extract was centrifuged for 2 min at maximum speed and 1.5 μl supernatant used per PCR reaction.

### PCR

PCR was used to genotype embryos and enable adult zebrafish sequencing. PCRs were conducted according to manufacturer’s specifications with 2x ReddyMix by Thermo Scientific/USA, 5x Firepol Master Mix by SolisBioDyne/Estonia or Phusion High-Fidelity DNA Polymerase by New England BioLabs/USA. A diagnostic digest was performed using the *mwol* restriction enzyme (NEB) and CutSmart® Buffer (NEB) according to manufacturer’s instructions. 4% agarose gels with ethidium bromide staining were used to visualize DNA fragments on a transilluminator. PCR purification prior to sequencing was performed using the MinElute PCR Purification Kit (Qiagen/ Netherlands) or, if unspecific amplification had occurred, the MinElute Gel Extraction Kit (Qiagen/Netherlands), according to the manufacturer’s manuals.

### 3D cyst assays

Cells were grown to confluence, washed in PBS and trypsinized before centrifuging for 5 min at 1000 rpm and resuspended in small amount of medium (approximately 400 µl). Rat tail collagen (MDCKII) or matrigel (OX161c1) were used as the respective 3D matrices to induce cyst formation. Cysts were induced and cultured as previously described^45,50,51^. 5 µM forskolin served as a positive control. Dose response assays were performed for toxicity and efficacy in both cystic cell lines, toxicity was assessed by visual inspection with concentrations provoking cell death deemed toxic and excluded. The following concentrations were used; 10 µM for Pimpinellin, diclofenac and dibutylhydroxyanisole; 1 µM for androstandione; prenyletin; sphondin and trimethoxyflavone; 0.1 µM for hexamethylquercetagetin and 0.01 µM for Zinc pyrithione. The medium (with compound) was prepared on the day the cells were seeded in a quantity sufficing the entire assay. MDCK cells were grown at 37° in Gibco DMEM/F-12 medium with 10% FBS, 1% Penicillin/Streptomycin and 1% L-glutamine media whee as OX161c1 cells were grown at 33 °C in Gibco DMEM/F-12 medium with 5% Nu-Serum, 1% Penicillin/Streptomycin and 1% L-glutamine. Medium on the cells was exchanged every 2–3 days and the cysts imaged and analysed with ImageJ.

### Apoptosis and proliferation assays *in situ*

To assess apoptosis and proliferation in cystic cell culture, immunofluorescence was used with anti-cleaved caspase-3 (Cell Signalling Technologies) or Ki-67 (Dianova) primary antibodies. Lumen formation was visualized using an antibody to podocalyxin (gp135) or phalloidin. MDCK cells were exposed to compounds of interest for 10 days before rinsing with PBS and digestion for 10-minutes at 37 °C with 100 U/ml collagenase (Sigma-Aldrich). Cells were then fixed in 4% PFA for 60 minutes, quenched with 0.15 mol/l glycine (Sigma-Aldrich) and permeabilized for 60 minutes with 0.1% Triton X-100 (VWR). Cells were blocked with 5% BSA for 60 minutes before incubation with primary antibodies to Ki-67 or cleaved caspase-3 in 5% BSA for 24 hours at 4 °C. The cells were then washed for an hour four times before incubation with the secondary antibody, Alexa Fluor® 488 conjugated-antibody (Life technologies), for a further 24 hours at 4 °C. Further 1 hour washes were subsequently performed before a 1-hour incubation with 4′,6-diamidino-2-phenylindole (DAPI, Sigma-Aldrich) at a concentration of 200 ng/ml. After washing, cells were mounted for microscopy with Vectashield (Vector Laboratories). Imaging was performed using an Olympus inverted IX71 microscope and analyzed using ImageJ software. The percentage of cysts with more than 5 nuclei with cleaved caspase-3 staining and percentage of Ki-67 cells per cyst were used to calculate apoptosis and proliferation rates respectively.

### Fixing adult zebrafish

Adult zebrafish were culled and fixed in 4% paraformaldehyde for 3–4 days at room temperature. Fixed zebrafish were then washed with PBS and decalcified with 0.25 M EDTA at room temperature for 2–3 days before being immersed in 70% ethanol. Adult zebrafish were then paraffin embedded and sectioned using a microtome.

### Bioinformatics analysis

The original human microarray dataset was identified and downloaded from the Gene Expression Omnibus (GEO, http://www.ncbi.nlm.nih.gov/geo/) (ID: GSE7869) as raw CEL files. The array was performed using GPL570 (Affymetrix Human Genome U133 Plus 2.0 Array). The kidney samples included 5 polycystic kidneys and 3 non-cancerous cortical tissue samples from kidneys removed for renal cell carcinoma as described by Song *et al*.^46^. The 5 polycystic kidney samples were separated into minimally cystic tissue (MCT) (n = 5) and small (<1 ml, n = 5), medium (10–20 ml, n = 5) and large cysts (>50 ml, n = 3)^46^. For cystic cell micro array expression analysis, a previous dataset was identified and downloaded from the ArrayExpress data base (https://www.ebi.ac.uk/arrayexpress/) (ID: E-MTAB-4189). The cell culture micro arrays are derived from two non-cystic cell cultures and three cystic cell cultures as outlined by Streets *et al*.^45^. Individual gene expression analysis was performed using Transcriptome Analysis Console (TAC), Thermo Fisher Scientific. Where multiple probes targeted the same gene, the sum of probe intensities was used for analysis.

## Ethical approval

All zebrafish were maintained under standard conditions in UK Home Office–approved aquaria at the Bateson center aquaria in the University of Sheffield. All procedures adhered to Home Office legislation and are subject to Animal Welfare and Ethical Review Bodies.

## Supplementary information

Supplementary information.
