# Supplementary material for: A high throughput zebrafish chemical screen reveals ALK5 and non-canonical androgen signalling as modulators of the pkd2−/− phenotype
Source: Sci Rep. 2020 Jan 9;10:72. doi: 10.1038/s41598-019-56995-7 (PMC6952374; doi:10.1038/s41598-019-56995-7)
Supplement: Supplementary file 1 — Supplementary information. [file 41598_2019_56995_MOESM1_ESM.pdf]

A high throughput zebrafish chemical screen reveals ALK5 and non-canonical androgen signalling as modulators of the *pkd2*$^{-/-}$ phenotype

Authors: Metzner A [1,2,3, a], Griffiths JD[1, a], Streets AJ[1], Markham E[2, 3], Philippou T[2, 3], Van Eeden FJM[2, 3] *, Ong ACM [1, 2] *

Affiliations:
1 Kidney Genetics Group, Academic Unit of Nephrology, Department of Infection, Immunity and Cardiovascular Disease, University of Sheffield, Sheffield, UK
2 The Bateson Centre, University of Sheffield, Sheffield, UK.
3 Department of Biomedical Science, University of Sheffield, Sheffield, UK.
[a] equal contribution
*Authors for correspondence

Supplementary data

Supplementary Table S1
Supplementary Table S2
Supplementary Figure S1
Supplementary Figure S2
Supplementary Figure S3
Supplementary Figure S4
Supplementary Figure S5
Supplementary Figure S6
Supplementary Figure S7
Supplementary Figure S8
Supplementary Figure S9
Supplementary References

Effect on $pkd2^{-/-}$	Chemical class	Compound name	Additional information	CAS number
enhancer		norethynodrel	progestin, formerly used as component in oral contraception[1]	68-23-5
enhancer	steroid	5alpha-androstan-3,17-dione	androgen, precursor of testosterone and estrogen, can function as bypass to anti-testosterone cancer treatment[2]	846-46-8
enhancer		epiandrosterone	androgen, precursor of testosterone and estrogen, non-competitive $GABA_A$ antagonist[3]	481-29-8
enhancer		prenyletin	isolated from *Ptaeroxylon obliquum*, antifungal[4]	15870-91-4
enhancer	coumarin	sphondin	isolated from *Heracleum maximum* root (mp 191-192 °C), Cox2 expression inhibition and PGE2 release inhibitor[5], antimycobacterial[6]	483-66-9
enhancer		pimpinellin	isolated from *Heracleum maximum* root (mp 118-119 °C), $GABA_A$ modulator[7], antimycobacterial[6]	131-12-4
enhancer		5,7,4'-trimethoxyflavone	isolated from *Cassia siamia* and *Citrus reticulata*, antioxidant[8], antihyperglycemic[9], adipocyte hypertrophy suppressor[10]	5631-70-9
enhancer	flavonoid	hexamethylquercetagetin	isolated from *Citrus spp*, antifungal, quercetin (very similar) inhibits EGFR pathway and prevents prostate cancer progression[25]	1251-84-9
enhancer	carboxylic ester	xanthoxylin	antifungal[16]	90-24-4
enhancer	chalcone	2',4'-dihydroxychalcone	isolated from *Flemingia chappar, Ceratiola ericoides, Acacia neovernicosa* and *Flourensia spp*, Cox2 inhibition[17], cell division inhibitor[18], induction of apoptosis[19], antileishmanial[20]	1776-30-3
repressor	phenylacetate	diclofenac sodium	NSAID, preferential Cox2/PGE2 inhibitor, L-type calcium channel inhibitor[21]	15307-79-6
repressor	pyridine	pyrithione zinc	antifungal, antibacterial, active ingredient in anti-dandruff shampoo, treatment for hidredenitis suppurativa[22] and seborrhoeic dermatitis[23]	13463-41-7
repressor	anisole	2,5-di-t-butyl-4-hydroxyanisole	3,5-di-t-butyl-4-hydroxyanisole (very similar) described to have Calcium antagonistic properties[24]	1991-52-2

Supplementary Table S1. Spectrum library hit compounds identified from zebrafish screen.

List of chemical compound zebrafish screen 'hits', their effect on *pkd2*$^{-/-}$ curvature phenotype, chemical class, CAS number and further information. CAS chemical library compounds are available to view at the spectrum chemical website: https://www.spectrumchemical.com/OA_HTML/Spectrum_CasNumLanding.jsp?minisite=10020&respid=22372. List of Spectrum library hit compounds, their effect on *pkd2*$^{-/-}$ curvature phenotype, the chemical class, CAS number and further information. 1 https://pubchem.ncbi.nlm.nih.gov/compound/norethynodrel, 2 [1-3], 3 [4,5], 4 [6], 5 [7], 6 [8], 7 [9], 8 [10], 9 [11], 10 [12], 11 [13], 12 [14], 13 [15], 14 [16], 15 [17,18], 16 [19], 17 [20], 18 [21], 19 [22], 20 [23], 21 [24], 22 [25], 23 [26], 24 [27-29], 25 [30], 26 [31].

Name	Referred to as	Chemical name	Most inhibited target known	Additional information
GW785804X	PKIS_04	4-(4-fluorophenyl)-5-(1,5-naphthyridin-2-yl)-1,3-thiazol-2-amine	KDR	originally designed against ALK5<br>ALK5 Binding – 0.042 μM<br>TGFβ Cellular Assay – 0.032 μM[1]<br>TGFBR1 inihitor[2], ALK5 inhibitor[3]
GW780159X	PKIS_59	4-(3-chlorophenyl)-5-(1,5-naphthyridin-2-yl)-1,3-thiazol-2-amine	KDR	originally designed against ALK5<br>ALK5 Binding – 0.023 μM<br>TGFβ Cellular Assay – 0.016 μM[1]<br>ALK5 inhibitor[3]
SB-698596-AC	PKIS_96	N-[5-(2,3-difluorophenyl)-1H-pyrazolo[3,4-c]pyridazin-3-yl]-2-(1-ethylpiperidin-4-yl)acetamide; 2,3-dihydroxybutanedioic acid	GSK3B	originally designed against GSK3A<br>GSK3 inhibitor[4]
GW682841X	PKIS_41	2-(4-propan-2-ylphenyl)-4-(5-pyridin-2-yl-1H-pyrazol-4-yl) pyridine	MAP4K4	originally designed against ALK5<br>ALK5 Binding – 0.032 μM[1]<br>ALK5 inhibitor[5]

Supplementary Table S2. PKIS repressors of *pkd2* phenotype identified from screen. List of PKIS repressor compounds with significant effect on *pkd2* curvature including name, referred name, kinase the compound was designed against, most potent known target and further information. 1 [32], 2 [33], 3 [34], 4 [35], 5 [36].

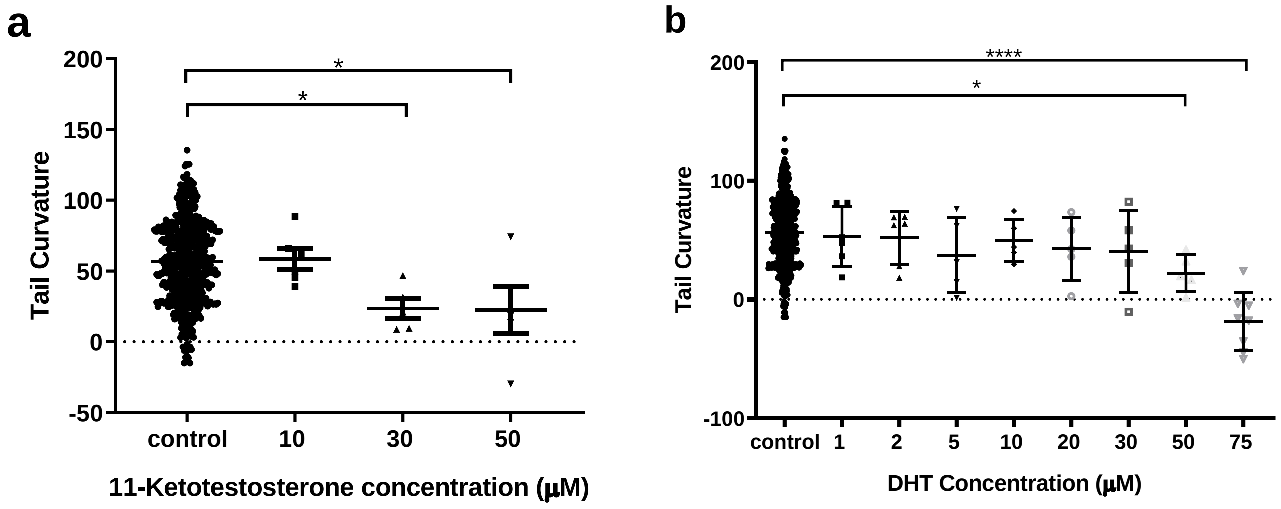

Supplementary Figure S1. Additional steroid compound testing

Effects of *pkd2* embryo exposure to the most potent zebrafish androgen, 11-KT (a) and the most potent human androgen DHT (b) on tail curvature. Results shown as mean ± SEM, Significances via one-way ANOVA with Dunnett's multiple comparisons; ****: $p \leq 0.0001$, ***: $p \leq 0.001$, **: $p \leq 0.01$, *: $p \leq 0.05$ and non-significant (ns): $p > 0.05$.

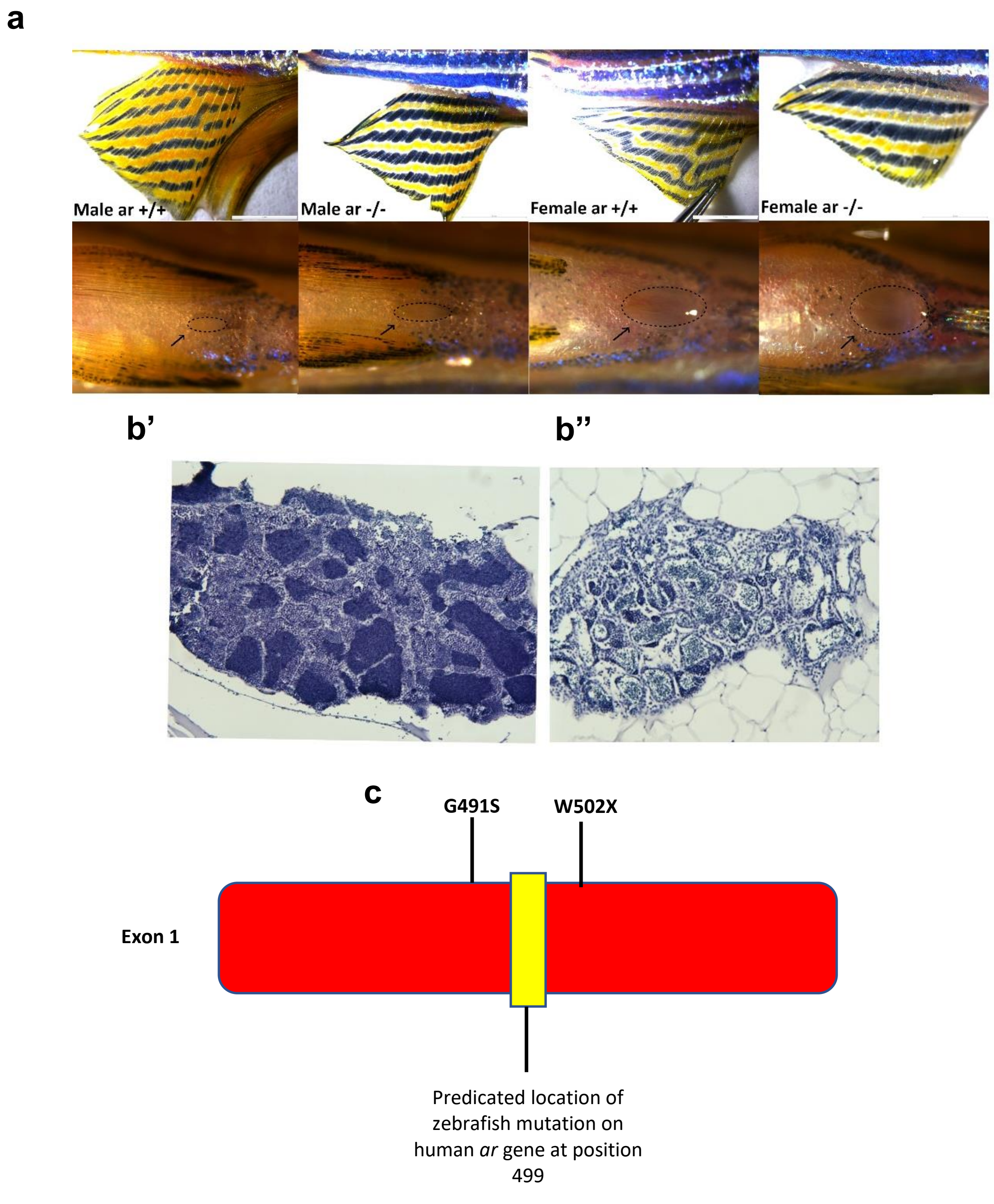

Supplementary Figure S2. Confirmation of ar loss-of-function phenotype
Images were obtained of male and female *ar*$^{-/-}$ and sibling adult zebrafish with anal fins (a). Female adult zebrafish and *ar*$^{-/-}$ male zebrafish have a pale anal fin compared to the yellow fin on the sibling male adult. Adult zebrafish genital papilla in female and male siblings and *ar*^{sh516}. Adult females have a protruding external genital papilla whilst males have withdrawn genital papilla in both *ar*^{sh516} and siblings. This demonstrates that despite female overall appearances, some *ar*^{sh516} mutant adults were in fact feminised males as previously reported[37-39]. (b) Histology showing the previously reported[37-39] disrupted and shrunken testicular architecture in the *ar* mutant zebrafish (b') compared to siblings (b''). Testes were

more withdrawn into the abdomen in mutants. (c) Analysis of the human *ar* mutation database[40] (available at http://androgendb.mcgill.ca/) showed that comparable mutations in human. The predicted location of our *ar* mutation is at 499 as indicated by the yellow box. Both of the closest mapped human mutations (W502X at position 502 and G491S at position 491) lead to complete androgen insensitivity syndrome[40]. Taken together these data show our mutant is a true null for AR function.

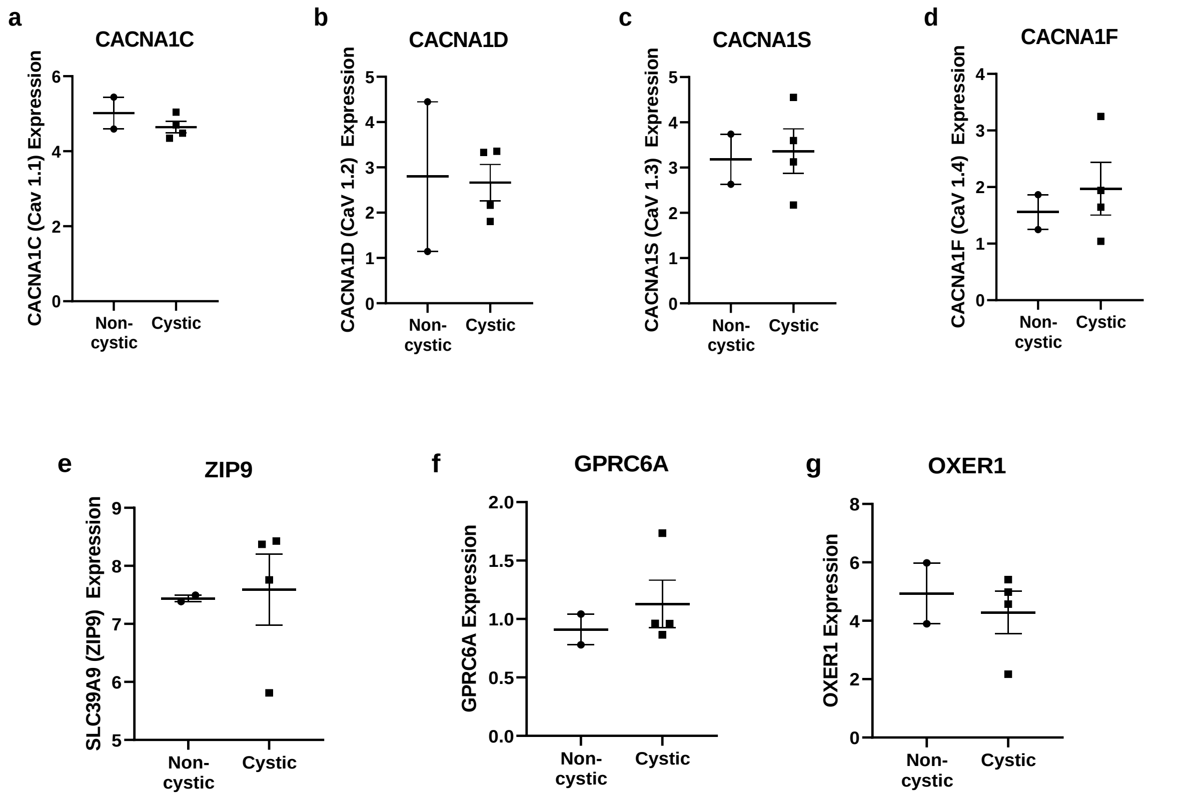

Supplementary Figure S3. Differential expression of LTCC subunits and candidate non-canonical androgen receptors in cystic cell cultures

Gene expression of CaV1.1 (a), CaV1.2 (b), CaV1.3 (c), CaV1.4 (d), ZIP9 (e), GPRC6A (f) and OXER1 (g) in cystic and non-cystic cell cultures. Expression profiles were dervived from published and publically available microarray data downloaded from the ArrayExpress data base (https://www.ebi.ac.uk/arrayexpress/) (ID: E-MTAB-4189). Results shown as mean expression ± SEM. Significances via one-way ANOVA with Dunnett's multiple comparisons; ****: $p \leq 0.0001$, ***: $p \leq 0.001$, **: $p \leq 0.01$, *: $p \leq 0.05$ and non-significant (ns): $p > 0.05$.

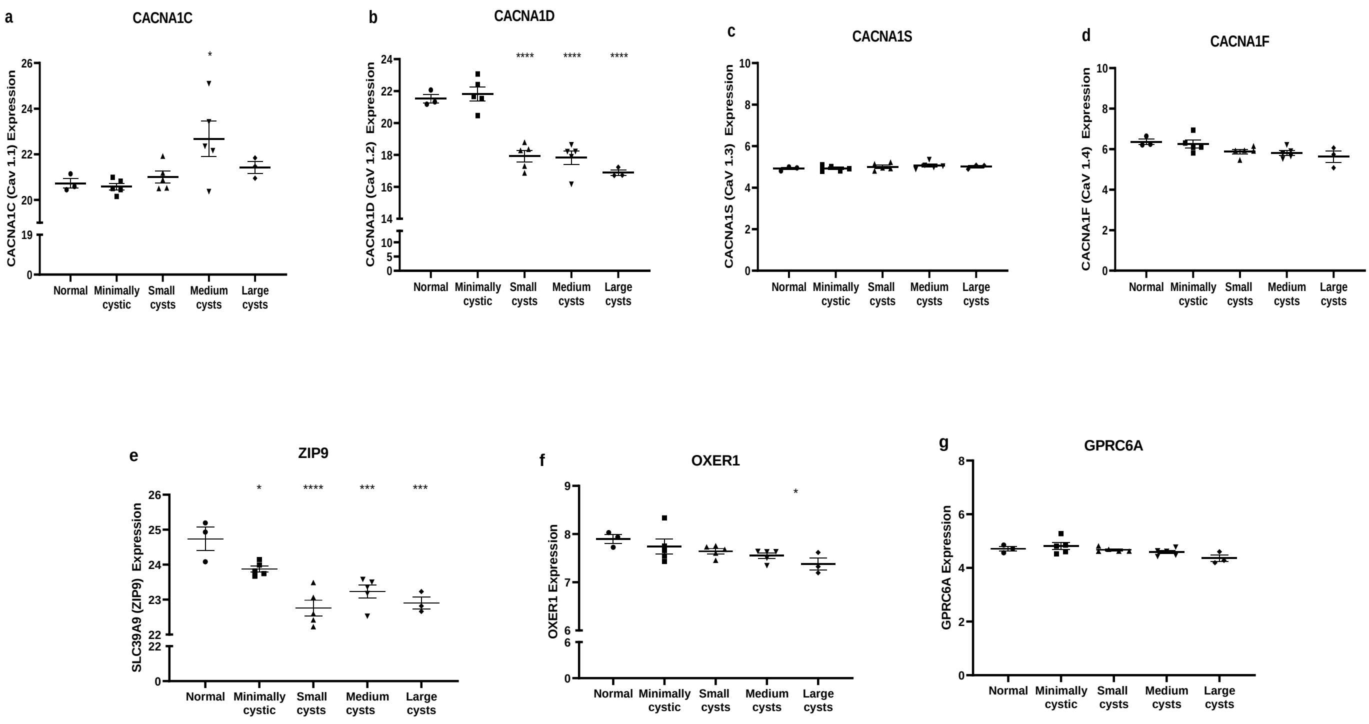

Supplementary Figure S4. Differential expression of LTCC subunits and candidate non-canonical androgen receptors in human kidneys

Gene expression of CaV1.1 (a), CaV1.2 (b), CaV1.3 (c), CaV1.4 (d), ZIP9 (e), GPRC6A (f) and OXER1 (g) in normal, minimally cystic kidney tissue and cysts in ADPKD patients. Samples were derived from 5 polycystic kidney samples and 3 non-polycystic kidney samples. Polycystic kidney tissues were separated into minimally cystic, small cysts (<1ml), medium cysts (10-20ml) and large cysts (>50ml). Expression profiles were derived from published and publically available microarray data (NCBI-GEO accession number: GSE7869). Results shown as mean expression ± SEM. Significances via one-way ANOVA with Dunnett's multiple comparisons; ****: $p \leq 0.0001$, ***: $p \leq 0.001$, **: $p \leq 0.01$, *: $p \leq 0.05$ and non-significant (ns): $p > 0.05$.

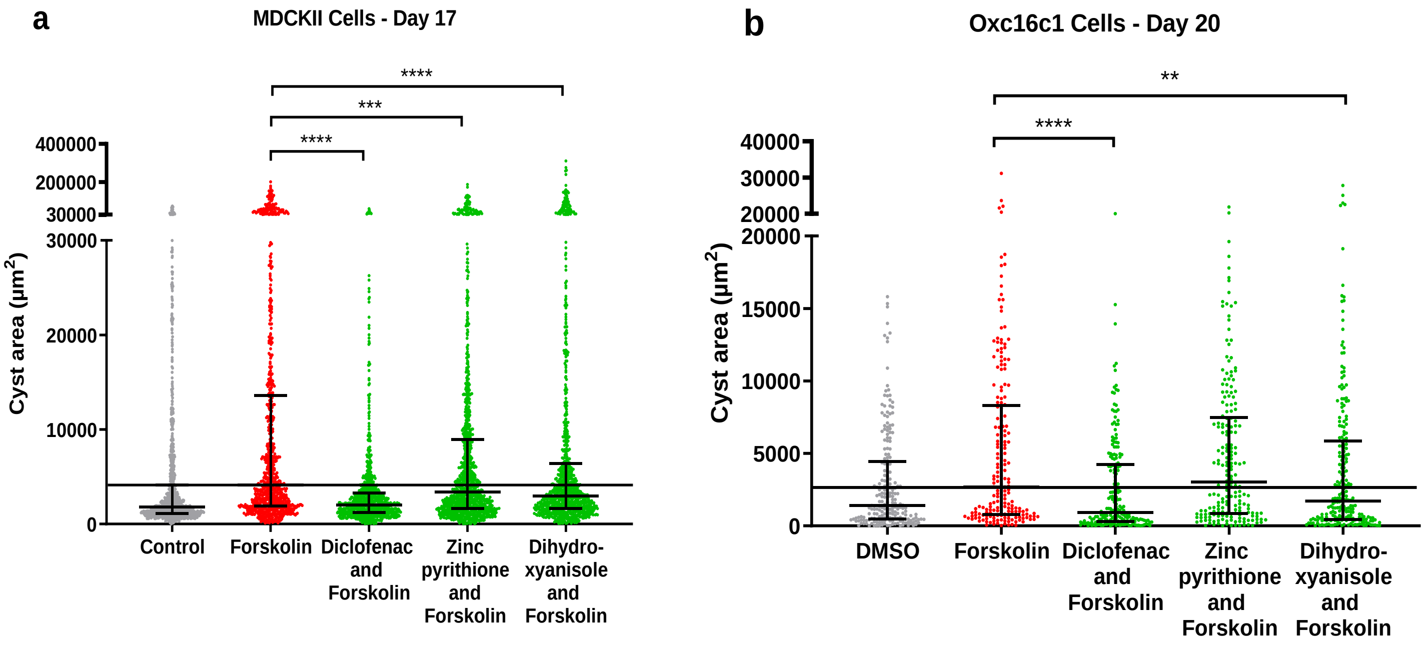

Supplementary Figure S5. Co-exposure of identified repressor compounds with Forskolin in cystic cell culture

Cyst area of MDCKII (a) and Ox161c1 (b) cells after 17 and 20 days of compound exposure respectively to repressor compounds identified in the zebrafish screen and forskolin. Chemical classes as indicated, concentrations determined via prior dose response assays to exclude toxicity. Results represented by median +/- IQR. P values represented by * (****: $p \leq 0.0001$, ***: $p \leq 0.001$, **: $p \leq 0.01$, *: $p \leq 0.05$ and non-significant (ns): $p > 0.05$.). Median of DMSO baseline indicated with black line and significances via Kruskal-Wallis test with Dunn's multiple comparison.

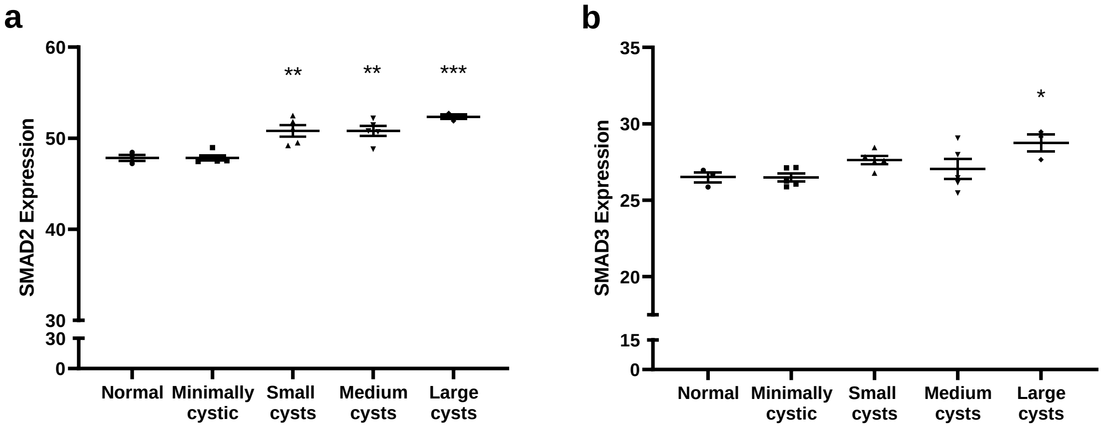

Supplementary Figure S6. Differential expression of SMAD2 and SMAD3 in ADPKD and control non-ADPKD human kidney tissue

Gene expression of SMAD2 (a) and SMAD3 (b) in normal, minimally cystic kidney tissue and cysts in ADPKD patients. Samples were derived from 5 polycystic kidney samples and 3 non-polycystic kidney samples. Polycystic kidney tissues were separated into minimally cystic, small cysts (<1ml), medium cysts (10-20ml) and large cysts (>50ml). Expression profiles were derived from published and publically available microarray data (NCBI-GEO accession number: GSE7869). Results shown as mean expression ± SEM. Significances via one-way ANOVA with Dunnett's multiple comparisons; ****: $p \leq 0.0001$, ***: $p \leq 0.001$, **: $p \leq 0.01$, *: $p \leq 0.05$ and non-significant (ns): $p > 0.05$.

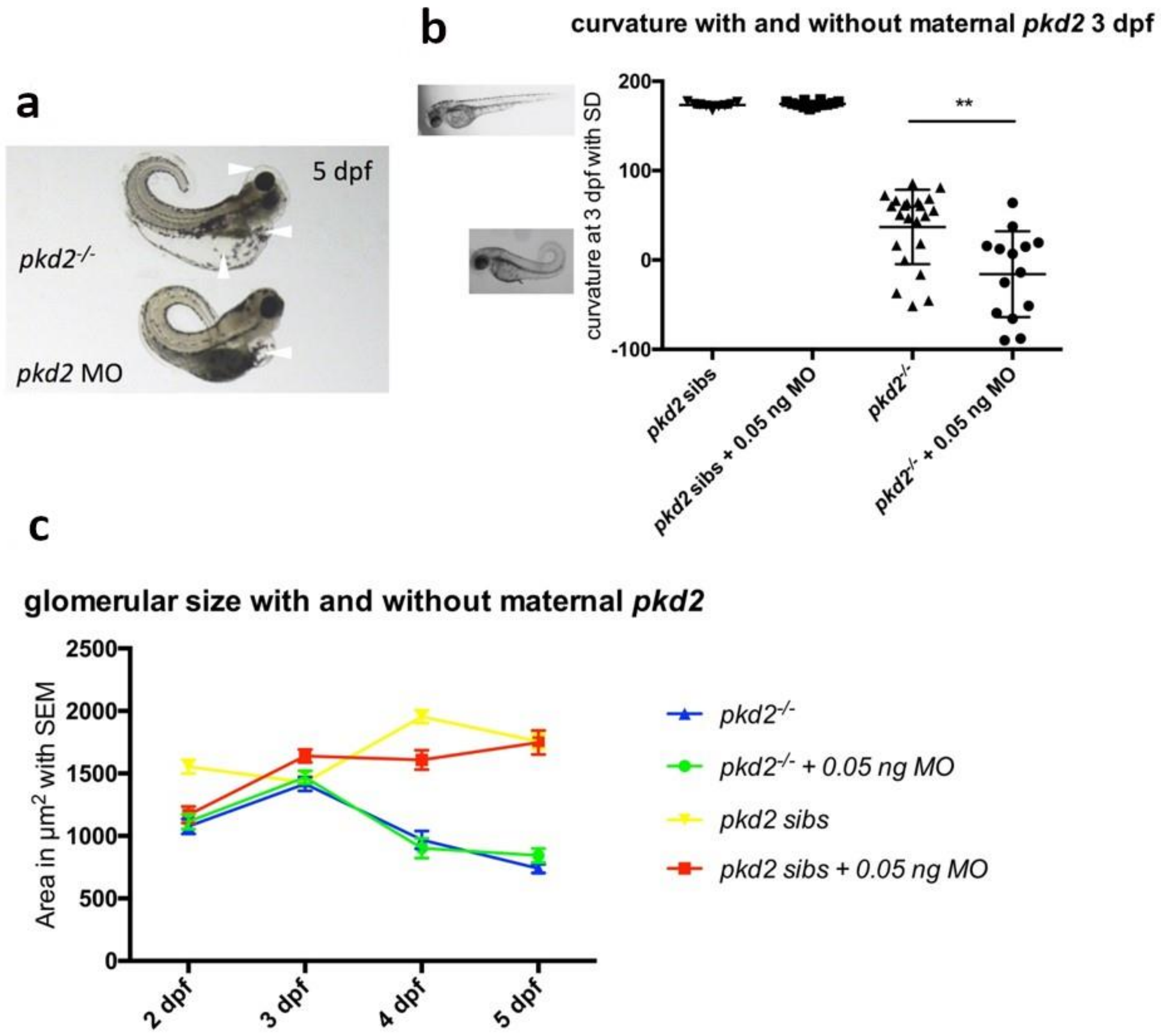

Supplementary Figure S7. The effects of maternal pkd2 mRNA on the *pkd2$^{-/-}$* embryo phenotype

(a) *pkd2* mutant and morphant embryos at 5 dpf, arrowheads indicating oedema. (b) Curvature severity comparing siblings, *pkd2* morpholino injected siblings, *pkd2$^{-/-}$* and *pkd2* morpholino injected *pkd2$^{-/-}$* embryos at 3 dpf. Significance via unpaired t-test; **: $p \leq 0.01$, *: $p \leq 0.05$ and non-significant (ns): $p > 0.05$. (c) Glomerular dilation of siblings, *pkd2* morpholino injected siblings, *pkd2$^{-/-}$* and *pkd2* morpholino injected *pkd2$^{-/-}$* embryos at days 2, 3, 4 and 5. No significant differences via two-way anova.

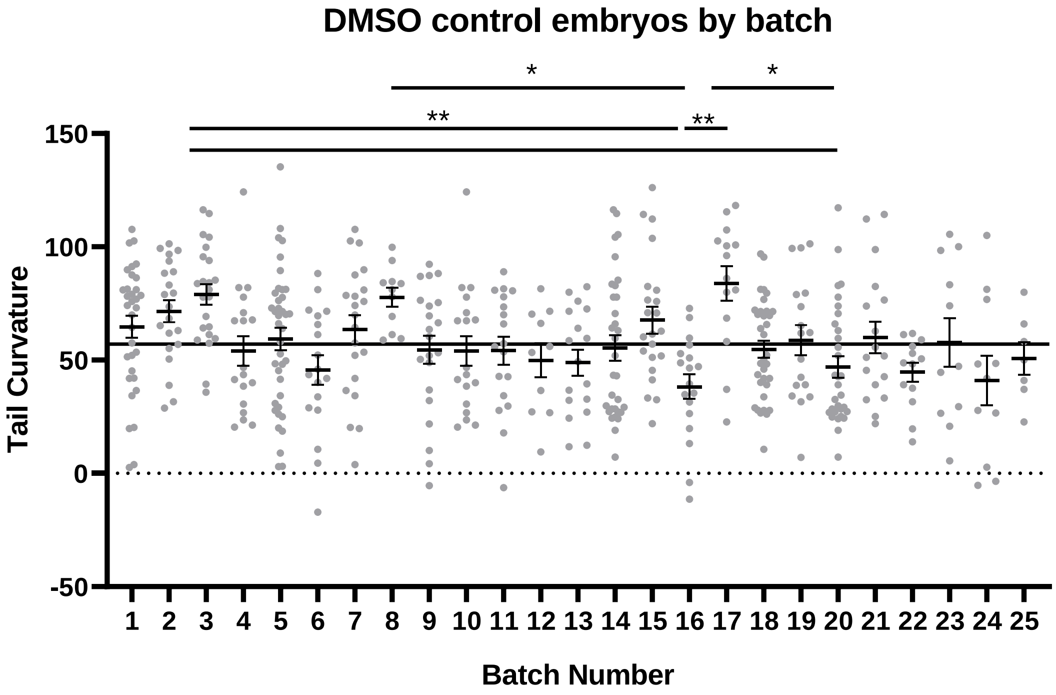

Supplementary Figure S8. DMSO control embryos from testing batchs
For final analysis of screen ‘hit’ compounds, a pooled group of DMSO control embryos were used. To ensure no significant variability between batches was present, the control groups from each batch were compared. The mean tail curvature angle was 58° (represented by the black line). The majority of batches showed no statistically significant difference between groups. However, there was some batch-to-batch variability. The groups with significant differences were limited to batches 3, 8, 16, 17 and 20. Significances via Kruskal-Wallis with multiple comparisons; ****: $p \leq 0.0001$, ***: $p \leq 0.001$, **: $p \leq 0.01$, *: $p \leq 0.05$ and non-significant (ns): $p > 0.05$.

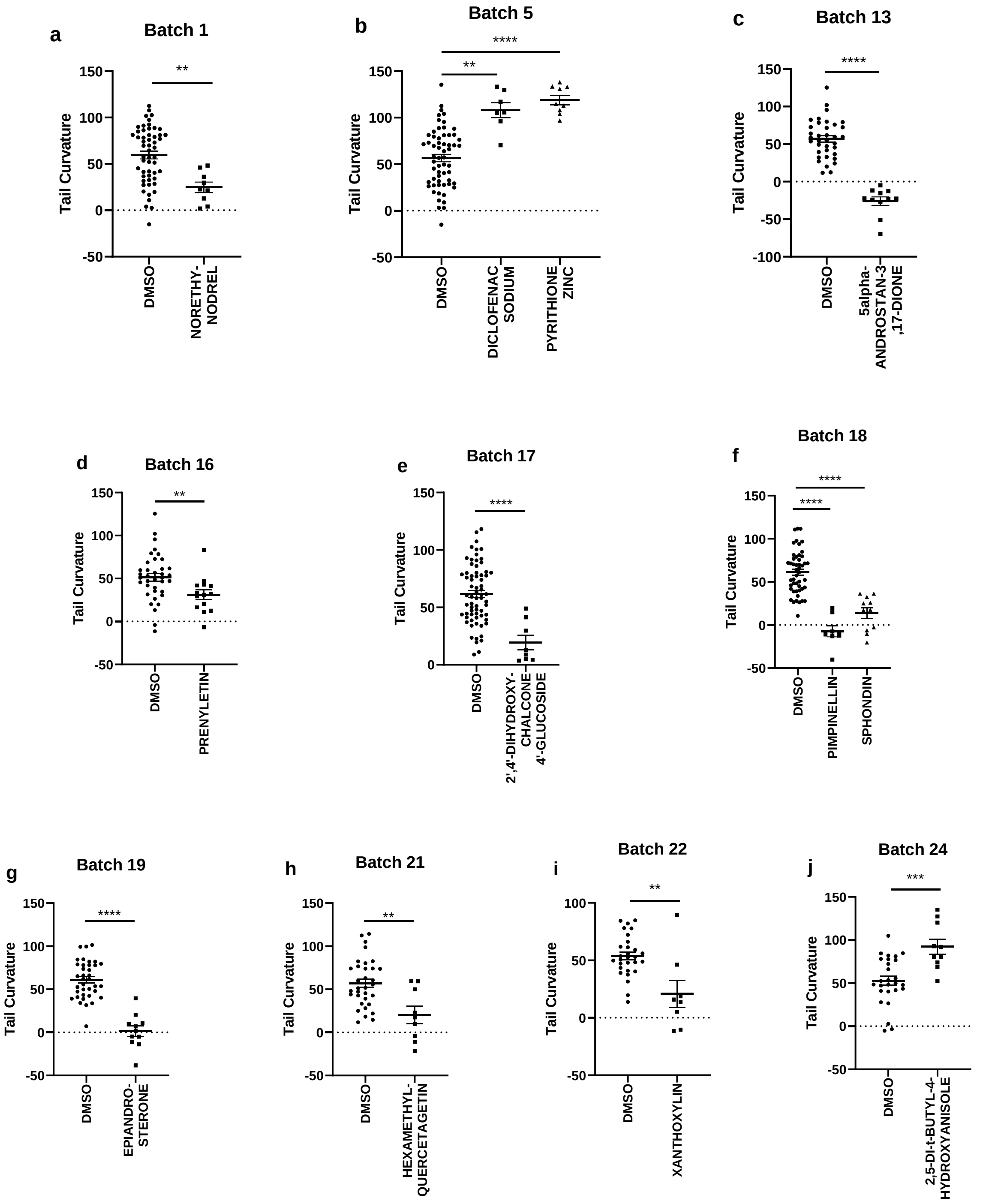

Supplementary Figure S9. Screen 'hits' compared only to control embryos from the same batch

The overall analysis of identified screen compounds used pooled DMSO control embryos from each batch. To confirm that the effects of hit compounds were not a result of batch-to-batch variability, exposed embryos were compared only with DMSO controls from the same batch. All identified 'hits' remained significant when during this analysis. Significances via Kruskal-Wallis with multiple comparisons (b & f) or Mann-Whitney tests; ****: $p \leq 0.0001$, ***: $p \leq 0.001$, **: $p \leq 0.01$, *: $p \leq 0.05$ and non-significant (ns): $p > 0.05$.

Supplementary References

1 Sharifi, N. The 5alpha-androstanedione pathway to dihydrotestosterone in castration-resistant prostate cancer. *J Investig Med* **60**, 504-507, doi:10.231/JIM.0b013e31823874a4 (2012).

2 Chang, K. H. *et al.* Dihydrotestosterone synthesis bypasses testosterone to drive castration-resistant prostate cancer. *Proc Natl Acad Sci U S A* **108**, 13728-13733, doi:10.1073/pnas.1107898108 (2011).

3 Li, Z. *et al.* Conversion of abiraterone to D4A drives anti-tumour activity in prostate cancer. *Nature* **523**, 347-351, doi:10.1038/nature14406 (2015).

4 Imamura, M. & Prasad, C. Modulation of GABA-gated chloride ion influx in the brain by dehydroepiandrosterone and its metabolites. *Biochem Biophys Res Commun* **243**, 771-775, doi:10.1006/bbrc.1998.8177 (1998).

5 Maninger, N., Wolkowitz, O. M., Reus, V. I., Epel, E. S. & Mellon, S. H. Neurobiological and neuropsychiatric effects of dehydroepiandrosterone (DHEA) and DHEA sulfate (DHEAS). *Front Neuroendocrinol* **30**, 65-91, doi:10.1016/j.yfrne.2008.11.002 (2009).

6 Stein, A. C., Alvarez, S., Avancini, C., Zacchino, S. & von Poser, G. Antifungal activity of some coumarins obtained from species of Pterocaulon (Asteraceae). *J Ethnopharmacol* **107**, 95-98, doi:10.1016/j.jep.2006.02.009 (2006).

7 Yang, L. L. *et al.* Effects of sphondin, isolated from Heracleum laciniatum, on IL-1beta-induced cyclooxygenase-2 expression in human pulmonary epithelial cells. *Life Sci* **72**, 199-213 (2002).

8 O'Neill, T., Johnson, J. A., Webster, D. & Gray, C. A. The Canadian medicinal plant Heracleum maximum contains antimycobacterial diynes and furanocoumarins. *J Ethnopharmacol* **147**, 232-237, doi:10.1016/j.jep.2013.03.009 (2013).

9 Singhuber, J., Baburin, I., Ecker, G. F., Kopp, B. & Hering, S. Insights into structure-activity relationship of GABAA receptor modulating coumarins and furanocoumarins. *Eur J Pharmacol* **668**, 57-64, doi:10.1016/j.ejphar.2011.06.034 (2011).

10 Bala, A., Chetia, P., Dolai, N., Khandelwal, B. & Haldar, P. K. Cat's whiskers flavonoid attenuated oxidative DNA damage and acute inflammation: its importance in lymphocytes of patients with rheumatoid arthritis. *Inflammopharmacology* **22**, 55-61, doi:10.1007/s10787-013-0193-5 (2014).

11 Takahashi, A. *et al.* Insulin resistance and low sympathetic nerve activity in the Tsumura Suzuki obese diabetic mouse: a new model of spontaneous type 2 diabetes mellitus and obesity. *Metabolism* **55**, 1664-1669, doi:10.1016/j.metabol.2006.08.007 (2006).

12 Okabe, Y. *et al.* Suppression of adipocyte hypertrophy by polymethoxyflavonoids isolated from Kaempferia parviflora. *Phytomedicine* **21**, 800-806, doi:10.1016/j.phymed.2014.01.014 (2014).

13 Erlund, I., Meririnne, E., Alfthan, G. & Aro, A. Plasma kinetics and urinary excretion of the flavanones naringenin and hesperetin in humans after ingestion of orange juice and grapefruit juice. *J Nutr* **131**, 235-241 (2001).

14 Liu, Y., An, W. & Gao, A. Protective effects of naringenin in cardiorenal syndrome. *J Surg Res* **203**, 416-423, doi:10.1016/j.jss.2016.03.003 (2016).

15 Wu, L. H. *et al.* Naringenin Suppresses Neuroinflammatory Responses Through Inducing Suppressor of Cytokine Signaling 3 Expression. *Mol Neurobiol* **53**, 1080-1091, doi:10.1007/s12035-014-9042-9 (2016).
16 Bao, L. *et al.* Naringenin inhibits proliferation, migration, and invasion as well as induces apoptosis of gastric cancer SGC7901 cell line by downregulation of AKT pathway. *Tumour Biol*, doi:10.1007/s13277-016-5013-2 (2016).
17 Alam, M. A., Kauter, K. & Brown, L. Naringin improves diet-induced cardiovascular dysfunction and obesity in high carbohydrate, high fat diet-fed rats. *Nutrients* **5**, 637-650, doi:10.3390/nu5030637 (2013).
18 Ikemura, M., Sasaki, Y., Giddings, J. C. & Yamamoto, J. Preventive effects of hesperidin, glucosyl hesperidin and naringin on hypertension and cerebral thrombosis in stroke-prone spontaneously hypertensive rats. *Phytother Res* **26**, 1272-1277, doi:10.1002/ptr.3724 (2012).
19 Vaz, Z. R., Filho, V. C., Yunes, R. A. & Calixto, J. B. Antinociceptive action of 2-(4-bromobenzoyl)-3-methyl-4,6-dimethoxy benzofuran, a novel xanthoxyline derivative on chemical and thermal models of nociception in mice. *J Pharmacol Exp Ther* **278**, 304-312 (1996).
20 Salas, A. L. *et al.* Biological activities of polyphenols-enriched propolis from Argentina arid regions. *Phytomedicine* **23**, 27-31, doi:10.1016/j.phymed.2015.11.007 (2016).
21 Xie, C., Sun, Y., Pan, C. Y., Tang, L. M. & Guan, L. P. 2,4-Dihydroxychalcone derivatives as novel potent cell division cycle 25B phosphatase inhibitors and protein tyrosine phosphatase 1B inhibitors. *Pharmazie* **69**, 257-262 (2014).
22 Sheng, Y., Zou, M., Wang, Y. & Li, Q. 2',4'-dihydroxychalcone, a flavonoid isolated from Herba oxytropis, suppresses PC-3 human prostate cancer cell growth by induction of apoptosis. *Oncol Lett* **10**, 3737-3741, doi:10.3892/ol.2015.3795 (2015).
23 Passalacqua, T. G. *et al.* The 2',4'-dihydroxychalcone could be explored to develop new inhibitors against the glycerol-3-phosphate dehydrogenase from Leishmania species. *Bioorg Med Chem Lett* **25**, 3564-3568, doi:10.1016/j.bmcl.2015.06.085 (2015).
24 Yarishkin, O. V. *et al.* Diclofenac, a Non-steroidal Anti-inflammatory Drug, Inhibits L-type Ca Channels in Neonatal Rat Ventricular Cardiomyocytes. *Korean J Physiol Pharmacol* **13**, 437-442, doi:10.4196/kjpp.2009.13.6.437 (2009).
25 Danesh, M. J. & Kimball, A. B. Pyrithione zinc as a general management strategy for hidradenitis suppurativa. *J Am Acad Dermatol* **73**, e175, doi:10.1016/j.jaad.2015.07.026 (2015).
26 Naldi, L. & Diphoorn, J. Seborrhoeic dermatitis of the scalp. *BMJ Clin Evid* **2015** (2015).
27 Fusi, F., Tzankova, V., Valoti, M., Pessina, F. & Sgaragli, G. 3,5-di-t-butyl-4-hydroxyanisole (DTBHA) activation of rat skeletal muscle sarcoplasmic reticulum Ca(2+)-ATPase. *Biochem Pharmacol* **62**, 1613-1619 (2001).
28 Sgaragli, G. P. *et al.* Calcium antagonist and antiperoxidant properties of some hindered phenols. *Br J Pharmacol* **110**, 369-377 (1993).
29 Fusi, F., Saponara, S., Gagov, H. & Sgaragli, G. Effects of some sterically hindered phenols on whole-cell Ca(2+) current of guinea-pig gastric fundus smooth muscle cells. *Br J Pharmacol* **132**, 1326-1332, doi:10.1038/sj.bjp.0703935 (2001).

30 Firdous, A. B. *et al.* Quercetin, a natural dietary flavonoid, acts as a chemopreventive agent against prostate cancer in an in vivo model by inhibiting the EGFR signaling pathway. *Food Funct* **5**, 2632-2645, doi:10.1039/c4fo00255e (2014).
31 Waheed, A. *et al.* Naringenin inhibits the growth of Dictyostelium and MDCK-derived cysts in a TRPP2 (polycystin-2)-dependent manner. *Br J Pharmacol* **171**, 2659-2670, doi:10.1111/bph.12443 (2014).
32 Elkins, J. M. *et al.* Comprehensive characterization of the Published Kinase Inhibitor Set. *Nat Biotechnol* **34**, 95-103, doi:10.1038/nbt.3374 (2016).
33 Singh, J. *et al.* Successful shape-based virtual screening: The discovery of a potent inhibitor of the type I TGF beta receptor kinase (T beta RI). *Bioorganic & Medicinal Chemistry Letters* **13**, 4355-4359, doi:10.1016/j.bmcl.2003.09.028 (2003).
34 Gellibert, F. *et al.* Identification of 1,5-naphthyridine derivatives as a novel series of potent and selective TGF-beta type I receptor inhibitors. *J Med Chem* **47**, 4494-4506, doi:10.1021/jm0400247 (2004).
35 Witherington, J. *et al.* 5-aryl-pyrazolo[3,4-b]pyridazines: potent inhibitors of glycogen synthase kinase-3 (GSK-3). *Bioorg Med Chem Lett* **13**, 1581-1584 (2003).
36 Gellibert, F. *et al.* Discovery of 4-{4-[3-(pyridin-2-yl)-1H-pyrazol-4-yl]pyridin-2-yl}-N-(tetrahydro-2H- pyran-4-yl)benzamide (GW788388): a potent, selective, and orally active transforming growth factor-beta type I receptor inhibitor. *J Med Chem* **49**, 2210-2221, doi:10.1021/jm0509905 (2006).
37 Crowder, C. M., Lassiter, C. S. & Gorelick, D. A. Nuclear Androgen Receptor Regulates Testes Organization and Oocyte Maturation in Zebrafish. *Endocrinology* **159**, 980-993, doi:10.1210/en.2017-00617 (2018).
38 Yu, G. *et al.* Zebrafish androgen receptor is required for spermatogenesis and maintenance of ovarian function. *Oncotarget* **9**, 24320-24334, doi:10.18632/oncotarget.24407 (2018).
39 Tang, H. *et al.* Fertility impairment with defective spermatogenesis and steroidogenesis in male zebrafish lacking androgen receptor. *Biol Reprod* **98**, 227-238, doi:10.1093/biolre/iox165 (2018).
40 Gottlieb, B., Beitel, L. K., Nadarajah, A., Paliouras, M. & Trifiro, M. The androgen receptor gene mutations database: 2012 update. *Hum Mutat* **33**, 887-894, doi:10.1002/humu.22046 (2012).
